# Kappa Light Chain-Restricted Multiple Myeloma with Biopsy-Proven Cast Nephropathy and Negative Bence–Jones Proteinuria: A Rare Clinical Presentation

**DOI:** 10.7759/cureus.96367

**Published:** 2025-11-08

**Authors:** Hamesh Gundala Raja, Manoj Sivakumar, Loveleen K Johal, Sumair Ali Khan, Fatimah Khan

**Affiliations:** 1 Internal Medicine, K.A.P. Viswanatham Government Medical College, Tiruchirappalli, IND; 2 Internal Medicine, St. George's University, True Blue, GRD; 3 Internal Medicine, Liaquat National Hospital and Medical College, Karachi, PAK; 4 Internal Medicine, Nishtar Medical University, Multan, PAK

**Keywords:** acute kidney injury, atypical presentation, cast nephropathy, light chain restriction, multiple myeloma, renal biopsy

## Abstract

Multiple myeloma (MM) is a malignant plasma cell disorder that commonly presents with anemia, bone pain, hypercalcemia, and renal impairment. We report the case of a 55-year-old male who presented primarily with acute kidney injury (AKI), pancytopenia, and systemic inflammation without the classical features of hypercalcemia or lytic bone lesions. Notably, urinary Bence-Jones proteins were negative despite markedly elevated serum kappa light chains and abnormal serum protein electrophoresis (SPEP). Bone marrow biopsy revealed modest plasmacytosis, while renal biopsy confirmed severe acute tubular injury with intratubular casts consistent with myeloma cast nephropathy. The patient required early initiation of hemodialysis and was subsequently managed with a bortezomib-based regimen, supportive care, and long-term planning for autologous stem cell transplantation (ASCT). This case underscores the importance of maintaining a high index of suspicion for MM in patients with unexplained AKI, highlights the indispensable role of renal biopsy in atypical presentations, and contributes to the growing recognition of variant clinical manifestations of this disease.

## Introduction

Multiple myeloma (MM) is a malignant plasma cell disorder characterized by clonal proliferation in the bone marrow and production of monoclonal immunoglobulins or free light chains [1]. It accounts for approximately 10% of hematologic malignancies and about 1% of all cancers worldwide. The incidence is higher in individuals of African ancestry compared to Caucasians, while Asians demonstrate lower rates. The disease most often presents in the sixth to seventh decade of life, with a slight male predominance [2].

The pathogenesis of MM is complex and involves genetic, environmental, and immunological factors. Clinically, patients present with features defined by the CRAB criteria: hyperCalcemia, Renal impairment, Anemia, and Bone lesions [3]. Renal dysfunction occurs in up to 40% of patients at diagnosis, with myeloma cast nephropathy being one of the most serious complications. This case report describes a 55-year-old male with kappa light chain-restricted MM complicated by biopsy-proven myeloma cast nephropathy and acute kidney injury (AKI) but with negative urinary Bence-Jones proteins. The objective is to highlight this atypical presentation and emphasize the importance of renal biopsy in establishing the diagnosis when conventional markers are inconclusive.

## Case presentation

A 55-year-old male presented to the nephrology outpatient clinic with progressive lower back pain for two weeks, decreased urine output for one week, and generalized weakness of the same duration. The pain began insidiously as mild discomfort while lifting objects and later occurred even with minimal activity, such as rising from bed. This was followed by oliguria, fever, and burning micturition, accompanied by marked fatigue, irritability, and reduced activity. There was no history of weight loss, night sweats, hemoptysis, or chronic joint pain. His past medical history was unremarkable, although there was a family history of diabetes mellitus and hypertension. On examination, his pulse was 78 beats per minute, blood pressure 150/90 mmHg, respiratory rate 16 per minute, and temperature 101°F. Pedal edema was present, while the remainder of the systemic examination was unremarkable. The initial workup revealed pancytopenia and AKI. Key hematology and biochemistry results are summarized in Table 1.

**Table 1:** Hematology and biochemistry profile at presentation. WBC: white blood cell, CRP: C-reactive protein, ESR: erythrocyte sedimentation rate, HbA1c: glycated hemoglobin (hemoglobin A1c)

Parameter	Result	Reference range	Interpretation
Hemoglobin	10.3 g/dL	13–18 g/dL	Anemia
WBC count	2.45 ×10⁹/L	4–11 ×10⁹/L	Leukopenia
Platelets	104 ×10⁹/L	150–400 ×10⁹/L	Thrombocytopenia
Urea	171 mg/dL	10–50 mg/dL	Elevated
Creatinine	9.34 mg/dL	0.5–0.9 mg/dL	Markedly elevated
Sodium	150 mmol/L	135–145 mmol/L	Hypernatremia
Potassium	3.4 mmol/L	3.5–5.0 mmol/L	Hypokalemia
Calcium	8.1 mg/dL	8.6–10 mg/dL	Hypocalcemia
Phosphate	6.5 mg/dL	2.5–4.5 mg/dL	Hyperphosphatemia
Uric acid	12.5 mg/dL	3.4–7.0 mg/dL	Hyperuricemia
CRP	10.5 mg/L	<5 mg/L	Elevated
ESR	20 mm/hr	0–25 mm/hr	Upper limit
HbA1c	6.36%	4.8–5.9%	Elevated

Urinalysis showed a clear appearance with a specific gravity of 1.010 and pH of 6.0; trace protein, 5-6 white blood cells per high-power field (WBCs/hpf), one to two red blood cells per high-power field (RBCs/hpf), and two to three epithelial cells per high-power field (hpf) were noted. Glycosuria, bilirubin, and ketones were negative. Importantly, urinary Bence-Jones proteins were absent. Despite controlled fluid intake (1.6-1.7 L/day) and careful hydration, the patient had persistent oliguria, signs of dehydration, and rising azotemia. The 24-hour urine output remained 900 mL. With the development of uremic features and no response to initial supportive measures (electrolyte correction, antihyperuricemic therapy, and empiric antibiotics), hemodialysis was initiated via an internal jugular central venous catheter on an alternate-day schedule, leading to improvement in uremic symptoms and partial biochemical stabilization.

A myeloma-directed evaluation demonstrated immunoglobulin (Ig) and light-chain abnormalities strongly suggestive of clonal plasma cell disease. Serum protein electrophoresis (SPEP) showed a sharp monoclonal spike in the gamma region with a reversed albumin-to-globulin ratio and a markedly elevated beta-2 microglobulin (β₂M) level. Targeted immunological and protein studies are summarized in Table 2. Inclusion of an SPEP densitometry trace as a figure would further strengthen the laboratory documentation in the final version.

**Table 2:** Immunological and protein studies.

Parameter	Result	Reference range	Interpretation
Serum IgG	63.7 g/L	5.4–18.2 g/L	Markedly elevated
Serum IgM	0.36 g/L	0.22–2.4 g/L	Low–normal
Serum Kappa light chain	915.14 mg/dL	122–437 mg/dL	Elevated
Serum Lambda light chain	19.11 mg/dL	62–231 mg/dL	Suppressed
κ/λ ratio	>47	0.26–1.65	Strong kappa restriction
β₂-microglobulin	60.65 mg/L	1–2.45 mg/L	Very high

**Figure 1:**
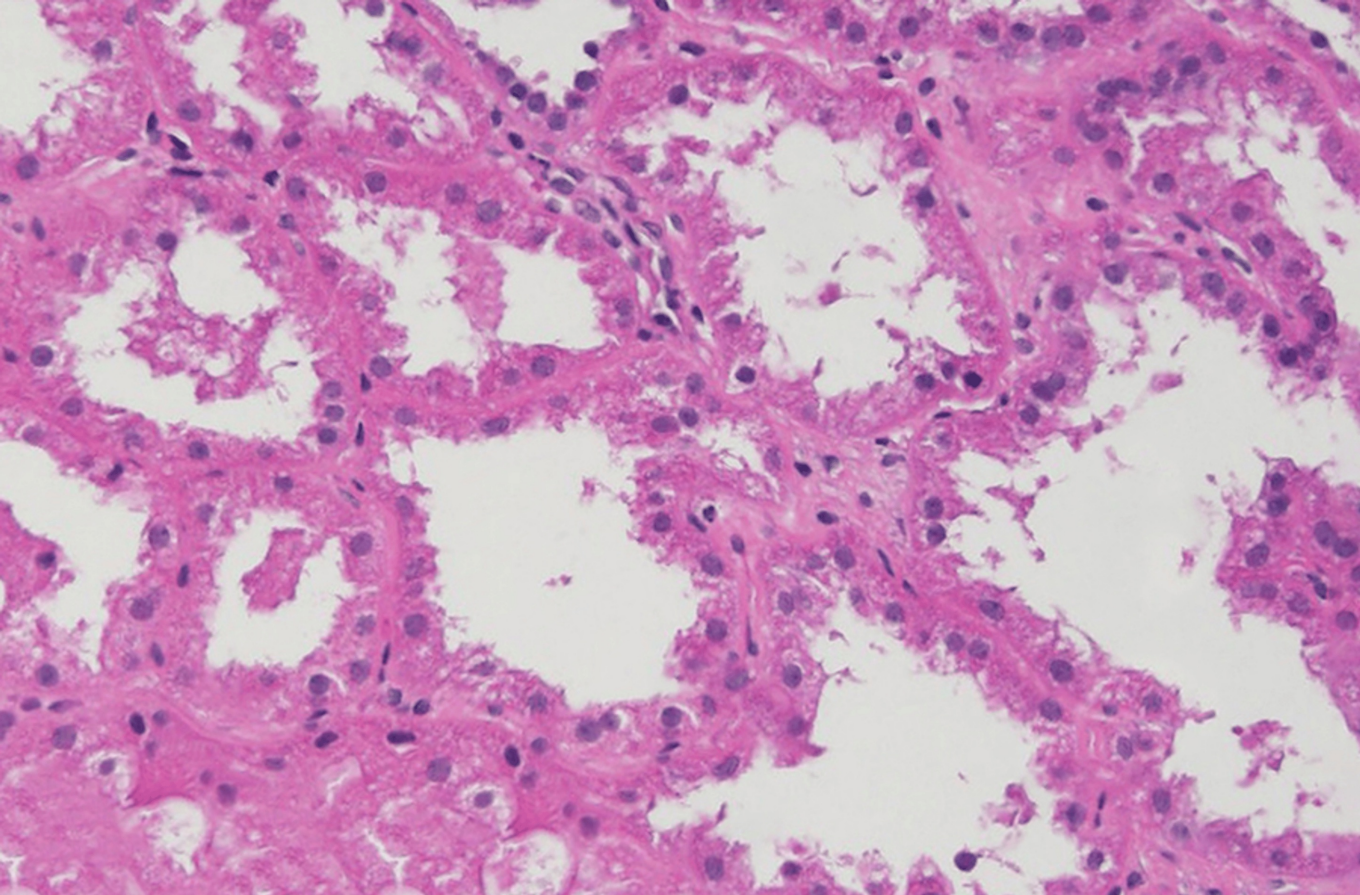
Renal biopsy showing severe acute tubular injury with tubular dilation, epithelial flattening, and loss of brush borders. The diagnosis of myeloma cast nephropathy in this case was based on comprehensive histopathologic assessment of the renal biopsy, including evaluation of multiple sections and immunofluorescence findings, and is not solely represented by this single illustrative image.

Peripheral blood smear confirmed pancytopenia with rouleaux formation. Bone marrow examination revealed a hypercellular marrow with approximately 10% mature-appearing plasma cells and preserved trilineage hematopoiesis, consistent with plasmacytosis in the appropriate clinical context. To delineate the cause of renal failure, a renal biopsy was performed once platelet counts were optimized and infection was excluded. Histology showed severe acute tubular injury with tubular dilatation, loss of brush border, epithelial cell dropout, and numerous fractured eosinophilic casts surrounded by macrophages, scattered neutrophils, and focal giant-cell reaction; interstitial fibrosis and tubular atrophy involved approximately 30-35% of the cortex with moderate interstitial nephritis and vascular changes (arteriosclerosis and arteriolar hyalinosis). The histology slide can be visualized in Figure 1. Immunofluorescence was negative for IgG, IgA, IgM, C1q, and C3, with mild granular kappa staining and negative lambda staining, supporting kappa-restricted cast formation. Inclusion of photomicrographs demonstrating classic “fractured” casts and kappa-restricted staining would be instructive for readers. While the representative image demonstrates acute tubular injury, the overall biopsy assessment identified intratubular proteinaceous casts with associated inflammatory reaction, and immunofluorescence showed kappa light-chain-restricted staining, supporting the diagnosis of myeloma cast nephropathy.

Integrating the clinical presentation, hematologic abnormalities, monoclonal IgG kappa paraprotein with an extreme κ/λ imbalance, elevated β₂-microglobulin, bone marrow plasmacytosis, and renal histopathology, the final diagnosis was kappa light chain-restricted IgG MM with biopsy-proven myeloma cast nephropathy, presenting as AKI on chronic kidney disease. Management comprised ongoing hemodialysis with strict fluid and electrolyte monitoring, initiation of a bortezomib-cyclophosphamide-dexamethasone regimen with a plan for lenalidomide maintenance pending response, and comprehensive supportive care, including platelet transfusions for pancytopenia, bisphosphonates for skeletal protection and bone pain, infection prophylaxis with antibiotics as indicated, and analgesia. The patient and family were counseled about the disease course, the need for close monitoring, and future therapeutic options; contingent on stabilization and response, assessment for autologous stem cell transplantation is planned at follow-up.

## Discussion

MM most frequently presents in elderly patients, with the majority of diagnoses made after the age of 65. The usual clinical picture includes bone pain, anemia, recurrent infections, hypercalcemia, and renal dysfunction, forming the basis of the CRAB criteria. In this case, the patient was relatively younger at 55 years and presented predominantly with AKI, rather than lytic bone lesions or hypercalcemia, making the initial clinical impression atypical. Such early and severe renal involvement is observed in only a subset of patients, and the absence of classical features often delays the consideration of MM in the differential diagnosis.

Renal impairment is reported in approximately 20-40% of patients at the time of diagnosis, but AKI due to myeloma cast nephropathy without detectable urinary Bence-Jones protein is highly unusual [4]. In this patient, urinalysis revealed no significant proteinuria, and Bence-Jones proteins were negative, despite markedly elevated serum kappa light chains. This discrepancy illustrates an important diagnostic challenge: the absence of urinary light chains cannot reliably exclude myeloma-related kidney disease. Reliance solely on non-invasive urine studies may therefore result in underdiagnosis or misclassification, underscoring the essential role of renal biopsy in confirming cast nephropathy, particularly in patients with unexplained renal failure. It is important to note that myeloma cast nephropathy is a clinicopathologic diagnosis derived from the integration of findings across multiple renal biopsy sections, immunofluorescence studies, and clinical correlation. A single published histologic image may not fully demonstrate all diagnostic features, and representative figures should be interpreted within the context of the complete biopsy evaluation. This reinforces why renal biopsy, rather than any single photomicrograph, remains the definitive tool for establishing the diagnosis in atypical cases.

The diagnostic complexity was further compounded by the presence of pancytopenia and elevated inflammatory markers, which initially suggested an infectious or autoimmune etiology. Peripheral smear and bone marrow examination revealed only 10% plasma cell infiltration, a level that falls short of the conventional thresholds often seen in symptomatic MM. However, the constellation of findings, including abnormal serum protein electrophoresis, a markedly elevated β₂-microglobulin level, and renal histology consistent with cast nephropathy, confirmed the diagnosis of kappa light chain-restricted MM [5]. This case, therefore, highlights the importance of a multimodal diagnostic approach integrating clinical suspicion, serum studies, bone marrow assessment, and, most critically, renal biopsy.

Management in this patient also deviated from typical pathways. Standard first-line interventions in myeloma-related renal dysfunction include aggressive hydration, correction of hypercalcemia, and initiation of disease-specific therapy. Despite these measures, renal function continued to deteriorate, necessitating the early initiation of hemodialysis. The coexistence of pancytopenia further complicated management, requiring transfusion support and heightened infection control [6]. Ultimately, a bortezomib-cyclophosphamide-dexamethasone regimen was initiated, aligning with international recommendations for patients with severe renal impairment, and supportive therapies such as bisphosphonates were employed for skeletal protection [7]. The patient’s case demonstrates that prompt initiation of dialysis and targeted therapy can stabilize systemic complications, laying the groundwork for future consideration of autologous stem cell transplantation once clinical status permits.

Taken together, this case underscores several important clinical lessons. First, MM must remain a differential diagnosis in middle-aged patients presenting with unexplained AKI, even when classical features are absent. Second, negative urinary Bence-Jones protein studies do not exclude myeloma-related renal disease, and renal biopsy is indispensable in confirming the diagnosis when laboratory findings are inconclusive. Finally, the combination of pancytopenia, negative urinary light chains, and biopsy-proven cast nephropathy represents a rare but clinically significant variant of disease presentation, contributing valuable insight to the existing literature on atypical manifestations of MM.

## Conclusions

This case illustrates an atypical presentation of MM in a relatively young patient who developed biopsy-proven cast nephropathy and AKI in the absence of urinary Bence-Jones proteins. The diagnostic process emphasizes that negative urine studies should not preclude further investigation when clinical suspicion remains high and that renal biopsy is indispensable in clarifying the etiology of unexplained renal dysfunction. Early recognition and prompt initiation of supportive care, dialysis, and disease-specific therapy are crucial to improving outcomes, particularly in cases complicated by pancytopenia and systemic inflammation. By documenting this rare constellation of findings, our report highlights the need for vigilance among clinicians when evaluating patients with acute kidney injury, reinforces the value of a multimodal diagnostic approach, and contributes to the growing body of evidence on the diverse renal manifestations of MM.
