# Correction: Kappa Light Chain-Restricted Multiple Myeloma with Biopsy-Proven Cast Nephropathy and Negative Bence–Jones Proteinuria: A Rare Clinical Presentation

**DOI:** 10.7759/cureus.c397

**Published:** 2026-01-26

**Authors:** Hamesh Gundala Raja, Manoj Sivakumar, Loveleen K Johal, Sumair Ali Khan, Fatimah Khan

**Affiliations:** 1 Internal Medicine, K.A.P. Viswanatham Government Medical College, Tiruchirappalli, IND; 2 Internal Medicine, St. George's University, True Blue, GRD; 3 Internal Medicine, Liaquat National Hospital and Medical College, Karachi, PAK; 4 Internal Medicine, Nishtar Medical University, Multan, PAK

The article has been corrected to clarify that the diagnosis of myeloma cast nephropathy was based on a comprehensive histopathologic evaluation of the renal biopsy, and not solely on the single representative image shown. While the published image demonstrates acute tubular injury, the full biopsy assessment identified intratubular proteinaceous casts with associated inflammatory reaction, and immunofluorescence revealed kappa light-chain–restricted staining, collectively supporting the diagnosis. The corrections emphasize that myeloma cast nephropathy is a clinicopathologic diagnosis requiring integration of findings across multiple biopsy sections, immunofluorescence studies, and clinical correlation, and that no single photomicrograph is sufficient to demonstrate all diagnostic features.

